# 

**Published:** 1889-11-23

**Authors:** 


### THE HOSPITAL

A WEEKLY JOURNAL OF
**Science, Medicine, Nursing & Philanthropy.**

SATURDAY, NOVEMBER 23rd, 1889.

**A Bishop's Ambition** - 113

**Words of Consolation**.—Thoughts - 114

**The Cesspools of Chichester: a Courageous Speech.** By the BISHOP OF CHICHESTER - 115

**Po ular Pastimes as Aids to Hospital Funds.** By A. W. WEBSTER - 116

**The Doctor's Pulpit.**—Physiological Candles - 117

**"Travelling for Health."** By DR. J. BOWLES DALY - 118

**Everybody's Page** - 119

**Notes and News** - 120

**Annotations.**—Fogs and Philosophers—A Rare Gift—Church Parades—Mild, but not safe - 122

**The Practitioner's Outlook and Retrospect:**

MEDICINE—Distribution of Disease - 123

SURGERY—Spinal Injuries - 124

THERAPEUTICS—Sulphonal - 124

SANITATION—Military Hygiene - 125

THE PRACTITIONER'S BOOKSHELF—The Skin Diseases of Infancy and Early Life - 125

**The Hospital Workshop.** XVI.—The Birmingham General Dispensary - 126

**The General Practitioners and Dr. Rentoul's Scheme** - 127

**Scraps and Gleanings** - 128

**EXTRA SUPPLEMENT—THE NURSING MIRROR.**

**En Passant.**—Intolerance—The Practical Asylums Board—Taunton Institution—Nursing in the North—Nursing Home at Bristol—Short Items - xxxiii
**A Hospital Boy** - xxxiv
**The Management of Children.**—III. Minor Ailments xxxv
**British Nurses' Association.**—A New Departure - xxxv
**Lectures, &c.** - xxxv
**Death in Our Ranks** - xxxvi
**Presentations—Notices—Notes and Queries** - xxxvi
**Appointments—Vacancies—Amusements** - xxxvi

